# Assessing HPV-related quality of life among Iranian women: psychometric evaluation of the Persian HPV-QoL

**DOI:** 10.1186/s41687-026-01177-z

**Published:** 2026-08-19

**Authors:** Leila AliAghdami, Sobhan Rahimi Esbo, Zeinab Maloum, Kowser Qaderi, Samira Barjasteh

**Affiliations:** 1 Student Research Committee, Health Research Institute, Babol University of Medical Sciences, Babol, Islamic Republic of Iran; 2 Department of Medical Education, School of Medicine, Tehran University of Medical Sciences, Tehran, Islamic Republic of Iran; 3 Social Determinants of Health Research Center, Department of Medical Sciences Education Development, Education Development Center, Babol, Islamic Republic of Iran; 4 Nursing & Midwifery Faculty, Urmia University of Medical Sciences, Urmia, Iran; 5 Fertility and Infertility Research Center, Health Technology Institute, Kermanshah University of Medical Sciences, Kermanshah, Islamic Republic of Iran; 6 Reproductive Health Researcher Center, Clinical Research Center, Urmia University of Medical Sciences, Urmia, Iran

**Keywords:** Human papillomavirus, Quality of life, Psychometric evaluation, Cross-cultural adaptation, Women’s health, Sexual health, Patient-reported outcomes

## Abstract

**Background:**

Human papillomavirus (HPV) infection can impose considerable psychological, emotional, and sexual challenges that adversely affect women’s health-related quality of life (HRQoL). Since experiences related to HPV-associated stigma, sexuality, and concerns about transmission are shaped by sociocultural factors, the availability of culturally relevant disease-specific assessment tools is essential for evaluating HPV-related quality of life in diverse populations.

**Objective:**

The present study was designed to translate the Human Papillomavirus Quality of Life Questionnaire (HPV-QoL) into Persian and examine its psychometric properties among Iranian women diagnosed with HPV infection.

**Methods:**

This methodological cross-sectional study was carried out between January and October 2024 and included 230 women with confirmed HPV infection. Of these, 200 participants were included in the psychometric evaluation, while an independent subsample of 30 participants completed the questionnaire twice for test–retest reliability assessment. The Persian adaptation of the HPV-QoL was developed through a forward–backward translation approach based on internationally accepted recommendations for cross-cultural instrument adaptation. The psychometric evaluation included assessment of face validity, content validity, structural validity, convergent and discriminant validity, reliability, measurement error, and interpretability. Responsiveness was not evaluated because of the cross-sectional study design. Structural validity was investigated using confirmatory factor analyses.

**Results:**

The Persian HPV-QoL showed acceptable psychometric characteristics and was well aligned with the cultural context of the target population. Content validity findings were satisfactory (CVI ≥ 0.78; Kappa ≥ 0.75). Confirmatory factor analysis supported a multidimensional structure consisting of four domains: general well-being, contagiousness, health, and sexuality. Following model refinement, the final model demonstrated acceptable overall fit. The questionnaire demonstrated high internal consistency for the overall scale (Cronbach’s α = 0.929) and acceptable test–retest reliability (ICC = 0.833). Significant positive correlations with the Sexual Quality of Life–Female (SQOL-F) questionnaire supported criterion-related convergent validity.

**Conclusion:**

The Persian version of the HPV-QoL provides preliminary evidence of satisfactory validity and reliability for assessing HPV-related quality of life among Iranian women. In addition to providing a culturally relevant patient-reported outcome measure, the study emphasizes the importance of considering psychosocial and sociocultural dimensions of HPV infection in clinical and research settings. The instrument may facilitate clinical assessment, future intervention studies, and cross-cultural research in women’s reproductive and sexual health.

## Introduction

Human papillomavirus (HPV) is among the most prevalent sexually transmitted viral infections worldwide and remains an important public health concern because of its established role in cervical cancer and other anogenital diseases [1–3]. Although most HPV infections are transient and asymptomatic, persistent infection with high-risk genotypes may result in precancerous lesions and invasive malignancies [1, 2]. Cervical cancer remains one of the leading causes of cancer-related morbidity and mortality among women globally, with disproportionately higher burdens observed in low- and middle-income countries [3]. Beyond its biomedical consequences, HPV infection has increasingly been recognized as an important psychosocial and reproductive health issue.

Growing evidence indicates that HPV infection can substantially affect women’s emotional well-being, sexual health, interpersonal relationships, and overall health-related quality of life (HRQoL) [4–8]. Women diagnosed with HPV commonly report anxiety, fear of cancer, shame, stigma, concerns regarding fertility and partner relationships, and distress associated with disclosure of the infection [8–11]. Importantly, these psychosocial consequences may persist even in women with minimal or no physical symptoms, highlighting the complex interaction between medical, emotional, sexual, and sociocultural dimensions of HPV infection. In many societies, particularly those with conservative sociocultural norms surrounding sexuality, sexually transmitted infections may also be associated with moral judgement and social stigma, thereby intensifying emotional distress and negatively influencing healthcare-seeking behaviors [12–14].

In the Iranian sociocultural context, perceptions of sexual and reproductive health are strongly influenced by cultural sensitivities and familial norms. Qualitative studies among Iranian women with HPV have demonstrated that diagnosis is frequently accompanied by feelings of self-blame, embarrassment, fear of rejection, concerns regarding marital stability, and anxiety about future intimate relationships [8, 12, 13]. Such experiences may adversely affect participation in screening programs, adherence to follow-up care, communication with healthcare providers, and attitudes toward reproductive and preventive healthcare interventions [12, 14]. Consequently, comprehensive assessment of HPV-related quality of life is essential for understanding the broader burden of HPV infection and for developing patient-centered psychosocial and reproductive healthcare strategies.

Despite growing recognition of the psychosocial impact of HPV infection, assessment of HPV-related HRQoL remains challenging. Many previous studies have relied on generic instruments designed to evaluate overall quality of life, anxiety, depression, or sexual functioning [15–18]. Although valuable, these instruments may not adequately capture HPV-specific experiences such as stigma, fear of viral transmission, concerns regarding oncological outcomes, and HPV-related sexual insecurity. Consequently, important dimensions of women’s lived experiences may remain insufficiently assessed.

To address this limitation, several HPV-specific quality-of-life instruments have been developed, including the CECA, CSFQ, HIP, and HPV-QoL questionnaires [19]. However, these instruments differ substantially in conceptual scope and clinical applicability. Some focus primarily on emotional burden or sexual functioning, whereas others provide broader multidimensional assessment. Among these instruments, the HPV-QoL questionnaire developed by Coronado et al. offers a comprehensive evaluation of HPV-related quality of life through domains assessing general well-being, sexuality, concerns regarding viral transmission, and health-related perceptions [20]. Previous investigations have reported acceptable measurement performance and potential clinical usefulness of the instrument in different populations [20, 21]. Nevertheless, psychometric instruments developed within one linguistic and cultural setting cannot be assumed to function equivalently across different populations. Adapting a psychometric instrument for use in another culture extends beyond direct linguistic translation and requires assessment of conceptual consistency, semantic clarity, contextual relevance, and psychometric stability within the target population. This issue is particularly important in HPV-related assessment because perceptions of stigma, sexuality, contagion, and interpersonal relationships are deeply shaped by sociocultural context. Despite the growing prevalence of HPV infection in Iran and increasing attention to women’s reproductive health needs, no culturally adapted and psychometrically evaluated Persian version of the HPV-QoL has previously been available.

Therefore, the present study aimed to translate, culturally adapt, and evaluate the psychometric properties of the Persian version of the HPV-QoL among Iranian women diagnosed with HPV infection. The availability of a culturally relevant and psychometrically sound instrument may facilitate more accurate assessment of HPV-related quality of life, improve identification of psychosocial distress, support evaluation of counseling and educational interventions, and contribute to more patient-centered reproductive and sexual healthcare.

## Methods

### Study design and participants

This cross-sectional methodological study was conducted to translate, culturally adapt, and psychometrically evaluate the Persian version of the Human Papillomavirus Quality of Life Questionnaire (HPV-QoL) among Iranian women diagnosed with HPV infection. The study was performed between January and October 2024 at university-affiliated gynecology and women’s health clinics in northwestern Iran.

Eligible participants were women aged 18 years or older with a confirmed diagnosis of HPV infection who were able to read and understand Persian and were willing to participate in the study. Women with pregnancy, recent malignancy, or severe psychiatric disorders were excluded. Participants were recruited using convenience sampling. Written informed consent was obtained from all participants prior to data collection.

### Instrument

The HPV-QoL questionnaire was originally developed by Coronado et al. to assess the impact of HPV infection and related conditions on women’s health-related quality of life [20]. The instrument consists of 15 items rated on a 5-point Likert scale ranging from 1 (“strongly agree”) to 5 (“strongly disagree”). The original questionnaire consists of 15 items organized into five first-order factors representing general well-being (psychological and social dimensions), contagiousness, health, and sexuality.

To assess criterion-related convergent validity, the Persian version of the Sexual Quality of Life-Female questionnaire (SQOL-F) was also administered. The SQOL-F contains 18 items scored on a 6-point Likert scale and has previously demonstrated satisfactory psychometric properties in Iranian populations [22, 23].

### Translation and cross-cultural adaptation

Permission to translate and use the HPV-QoL questionnaire was obtained from its original developer [20]. The translation procedure followed a five-step forward-backward methodology [24]. In the initial step, at least two bilingual translators fluent in both Persian and English independently translated the questionnaire items using a semantic translation strategy within a forward-translation framework. In the second step, a supervising translator compared the forward-translated versions, resolved any discrepancies, and produced a consolidated version based on them. Third, the consolidated version was back-translated into English by two Persian-English bilingual translators who had no prior knowledge of the original questionnaires. Fourth, an expert committee comprising three to four bilingual translators reviewed the forward, consolidated, and backward versions. This committee included a linguistics expert, a questionnaire translation specialist, a content expert familiar with the underlying concepts, and a coordinator. An expert committee reviewed the translated versions to evaluate semantic, conceptual, and contextual equivalence. The fifth and final stage involved a pre-test, during which the pre-final version was administered to the target population. The final back-translated version was subsequently reviewed by the original developer of the HPV-QoL (Coronado et al.) for final validation.

### Psychometric evaluation

Psychometric evaluation included assessment of face validity, content validity, construct validity, convergent and discriminant validity, reliability, measurement error, and interpretability. Responsiveness was not evaluated because of the cross-sectional study design.

### Face validity

Face validity was evaluated using qualitative and quantitative approaches. For qualitative assessment, the preliminary Persian version of the questionnaire was reviewed by women from the target population to evaluate item clarity, comprehensibility, relevance, and difficulty. Feedback obtained during this stage was used to refine the wording of several items.

Quantitative face validity was assessed using the item impact method. Participants rated the importance of each item on a 5-point Likert scale ranging from 1 (“not important”) to 5 (“very important”). Items with impact scores greater than 1.5 were considered acceptable [25].

### Content validity

Content validity of the Persian version of the HPV-QoL was examined using qualitative and quantitative approaches. For the qualitative assessment, the questionnaire was reviewed by 16 experts in midwifery and women’s health who had experience in questionnaire translation and psychometric evaluation. The experts evaluated the items regarding wording, grammatical structure, clarity, appropriateness, and scoring format. Revisions were made based on their recommendations.

Quantitative content validity was assessed using the Content Validity Ratio (CVR) and Content Validity Index (CVI). To calculate CVR, the expert panel rated the necessity of each item using a 3-point scale ranging from “not necessary” to “necessary.” CVR values were calculated according to Lawshe’s method [26], and values above 0.62 were considered acceptable for retention of items [27].

The Content Validity Index (CVI) was subsequently calculated for each item. The same panel of experts rated item relevance using a 4-point scale ranging from irrelevant to completely relevant. Based on established recommendations, item-level CVI values of 0.79 or higher were considered acceptable [28]. In addition, the Scale-level Content Validity Index based on universal agreement (S-CVI/UA) and the average Scale-level Content Validity Index (S-CVI/Ave) were calculated, with recommended thresholds of 0.80 and 0.90, respectively [29, 30].

To further evaluate agreement among experts beyond chance, modified kappa coefficients were calculated for each item based on the corresponding I-CVI values and probability of chance agreement. Kappa statistics were interpreted according to established recommendations, with values above 0.74 considered indicative of excellent agreement. According to recommendations by Polit [31] and Beck [32], items with acceptable kappa coefficients and item-level Content Validity Index (I-CVI) values of 0.78 or higher were considered excellent.

### Item analysis

Corrected item-total correlations were examined prior to factor analysis to assess the contribution of each item to the overall scale. Items with corrected item-total correlation coefficients below 0.32 were considered for additional evaluation [33].

### Construct validity

Construct validity was assessed using confirmatory factor analysis (CFA). A total of 200 women participated in this phase of the study.

CFA was conducted using maximum likelihood estimation to examine the structural validity of the extracted factor model. Model adequacy was evaluated using multiple goodness-of-fit indices, including the chi-square to degrees of freedom ratio (χ²/df), Root Mean Square Error of Approximation (RMSEA), Goodness-of-Fit Index (GFI), Adjusted Goodness-of-Fit Index (AGFI), Incremental Fit Index (IFI), Comparative Fit Index (CFI), Parsimony Comparative Fit Index (PCFI), and Standardized Root Mean Square Residual (SRMR). Recommended criteria reported in the psychometric literature were used to interpret model fit [34–36].

### Convergent and discriminant validity

Convergent and discriminant validity were examined using the Fornell–Larcker approach [37]. Convergent validity was considered acceptable when Average Variance Extracted (AVE) values exceeded 0.50 and Composite Reliability (CR) values were greater than 0.70. Discriminant validity was supported when the Maximum Shared Variance (MSV) for each construct was lower than the corresponding AVE value [38].

### Reliability, measurement error, and interpretability

Internal consistency was evaluated using Cronbach’s alpha coefficient and average inter-item correlation (AIC). Cronbach’s alpha values above 0.70 were considered indicative of acceptable internal consistency [39, 40].

Test–retest reliability was assessed using the intraclass correlation coefficient (ICC) in a subgroup of 30 participants who completed the questionnaire twice, with a two-week interval between administrations. Measurement error was assessed using the Standard Error of Measurement (SEM) and the Minimal Detectable Change (MDC). The Minimal Important Change (MIC) was also calculated to facilitate interpretation of measurement precision; however, responsiveness was not evaluated because of the cross-sectional test–retest design [41–44].

### Data analysis

Statistical analyses were performed using SPSS version 27 and AMOS version 24. Participants’ demographic characteristics are summarized in (Table 1). Prior to factor analysis, assumptions related to univariate and multivariate normality were examined using skewness, kurtosis, Mahalanobis distance, and Mardia’s coefficient [45, 46].

### Ceiling and floor effects

Floor and ceiling effects were examined to determine the distribution of responses across the scale. These effects were considered present when more than 15% of participants achieved the lowest or highest possible scores, respectively, indicating reduced variability and limited ability of the instrument to detect differences or changes in the measured construct [47].

### Scoring

The final Persian version of the HPV-QoL consisted of 15 items across four domains. Items were scored using a 5-point Likert scale ranging from 1 (“Strongly Agree”) to 5 (“Strongly Disagree”). Total raw scores ranged from 15 to 75, with higher scores reflecting better health-related quality of life. Raw scores were subsequently transformed to a standardized 0–100 scale to facilitate interpretation and comparison across domains.

## Results

### Participant characteristics

A total of 200 women with confirmed HPV infection participated in the psychometric evaluation phase of the study. The mean age of the participants was 33.60 ± 9.26 years. Participants’ demographic characteristics are summarized in Table 1.

**Table 1:** Demographic characteristics of the participants (*N* = 200)

Variable	Category	*n* (%)
Education	< Diploma	95 (47.5)
	≥ Diploma	105 (52.5)
Husband’s education	< Diploma	81 (40.5)
	≥ Diploma	119 (59.5)
Employment status	Employed	65 (32.5)
	Housewife	135 (67.5)
Husband’s occupation	Employed	90 (45.0)
	Freelance	110 (55.0)
Marital status	Single	36 (18.0)
	Married	164 (82.0)
Type of marriage	Temporary	62 (31.0)
	Permanent	138 (69.0)

### Face and content validity

During qualitative face validity assessment, minor modifications in wording and formatting were made based on participant feedback to improve item clarity and comprehensibility. Quantitative face validity findings showed that all items achieved impact scores above the acceptable threshold of 1.5, indicating satisfactory perceived importance.

Qualitative content validity assessment led to several linguistic and contextual refinements based on expert feedback to improve conceptual clarity and cultural appropriateness. In the quantitative evaluation, all items demonstrated acceptable validity indices, with CVR values ≥ 0.62, CVI values ≥ 0.78, and modified kappa coefficients ≥ 0.75. These findings indicated strong expert agreement regarding the relevance and necessity of the questionnaire items. In addition, scale-level content validity indices were satisfactory, with S-CVI/UA and S-CVI/Ave values of 0.867 and 0.989, respectively (Table 2).

**Table 2:** Content validity indices of the Persian version of the HPV-QoL

Item	CVR	CVI
Item 1	0.92	1.00
Item 2	1.00	1.00
Item 3	0.92	0.92
Item 4	0.83	1.00
Item 5	1.00	1.00
Item 6	1.00	1.00
Item 7	1.00	1.00
Item 8	1.00	1.00
Item 9	1.00	1.00
Item 10	1.00	1.00
Item 11	0.92	1.00
Item 12	0.92	1.00
Item 13	0.92	0.92
Item 14	0.92	1.00
Item 15	1.00	1.00

Abbreviations: CVR = Content Validity Ratio; CVI = Content Validity Index

### Construct validity

**Fig. 1:**
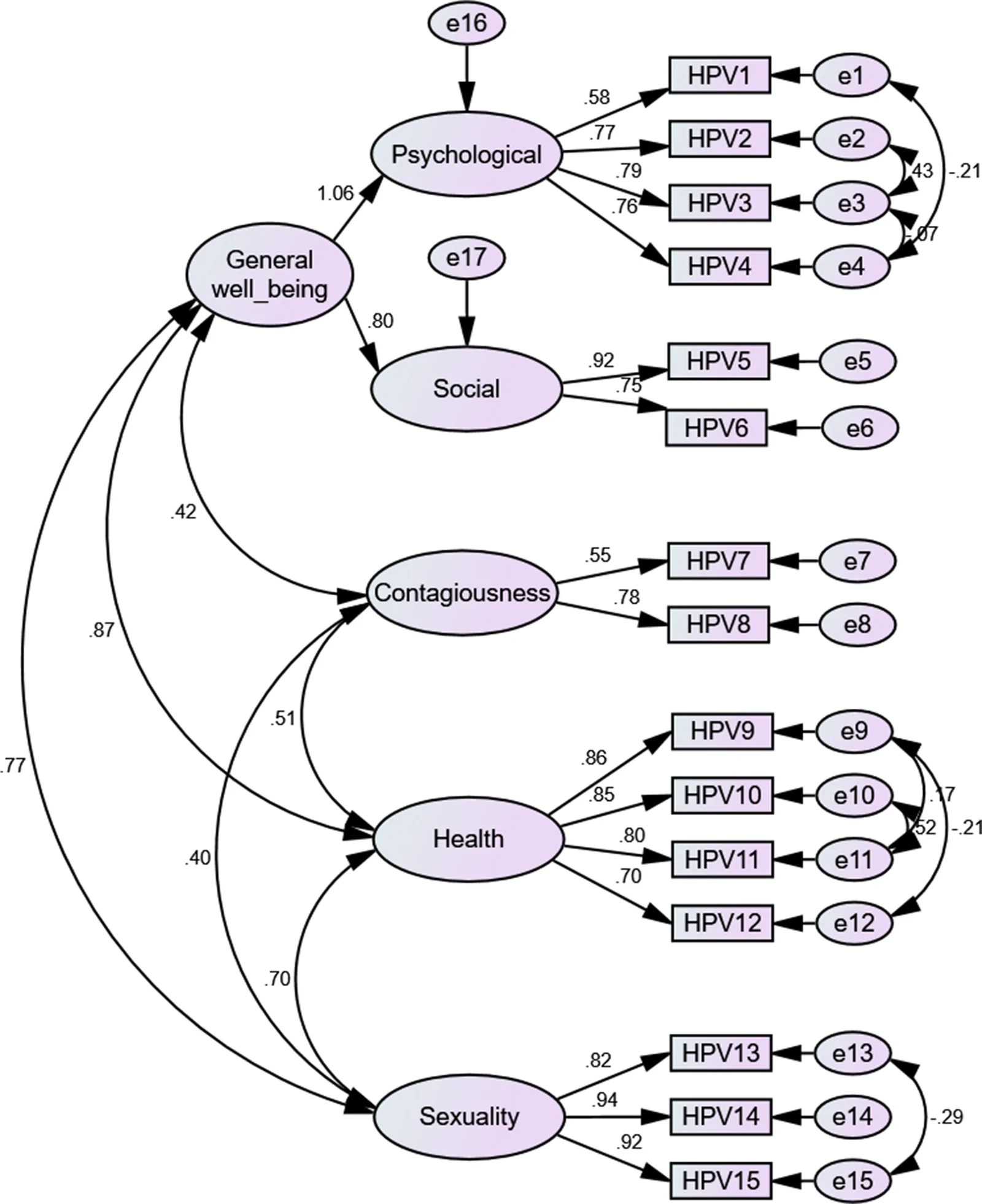
Confirmatory factor analysis based on the default model of the Persian version of the HPV-QoL scale

Initially, the original five-factor structure proposed by Coronado et al. (20) was evaluated (Fig. 1).The initial model demonstrated acceptable overall fit indices (Table 3). However, examination of the standardized factor loadings identified a loading greater than 1.0 within the General Well-being domain, suggesting possible multicollinearity and conceptual overlap between latent variables. Therefore, the model was re-specified based on both statistical evidence and conceptual considerations.

**Table 3:** Model fit indices for confirmatory factor analysis of the Persian HPV-QoL

Model	χ²	df	χ²/df	RMSEA	PNFI	PCFI	AGFI	GFI	IFI	NFI	CFI	SRMR
Initial five-factor model	195.015	75	2.600	0.090	0.651	0.673	0.828	0.892	0.943	0.911	0.942	0.061
Revised first-order model	207.952	76	2.736	0.093	0.655	0.678	0.818	0.885	0.937	0.905	0.937	0.067
Final second-order model	196.923	73	2.698	0.092	0.633	0.654	0.817	0.889	0.941	0.910	0.940	0.064

Abbreviations: RMSEA = Root Mean Square Error of Approximation; PNFI = Parsimonious Normed Fit Index; PCFI = Parsimony Comparative Fit Index; AGFI = Adjusted Goodness-of-Fit Index; GFI = Goodness-of-Fit Index; IFI = Incremental Fit Index; NFI = Normed Fit Index; CFI = Comparative Fit Index; SRMR = Standardized Root Mean Square Residual

The final Persian HPV-QoL retained four first-order domains. The allocation of the 15 questionnaire items to each domain is presented in (Table 4).

**Table 4:** Final domain structure and item allocation of the Persian HPV-QoL

Domain	Items
General well-being	Items 1–6
Contagiousness	Items 7–8
Health	Items 9–12
Sexuality	Items 13–15

**Fig. 2:**
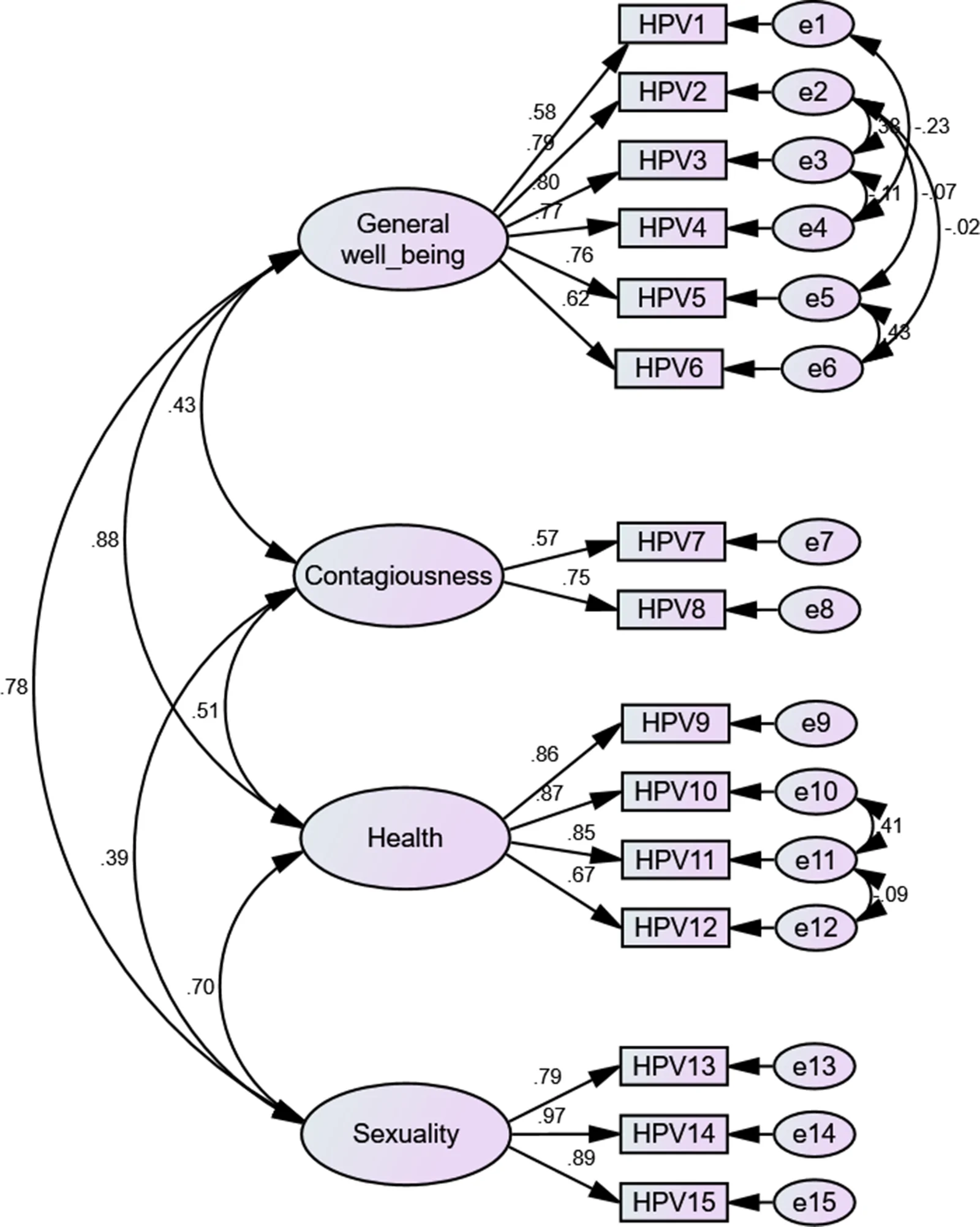
Confirmatory factor analysis of modified first-order model of the Persian version of the HPV-QoL scale

The revised model demonstrated improved structural coherence while retaining an acceptable overall fit to the data (Fig. 2; Table 3). Convergent and discriminant validity were subsequently evaluated using the Fornell–Larcker approach. The findings supported acceptable convergent validity for all latent constructs except the contagiousness domain (Table 5). In addition, discriminant validity analysis indicated considerable overlap between the General Well-being and Health-related constructs, suggesting limited distinction between these domains within the study sample.

**Fig. 3:**
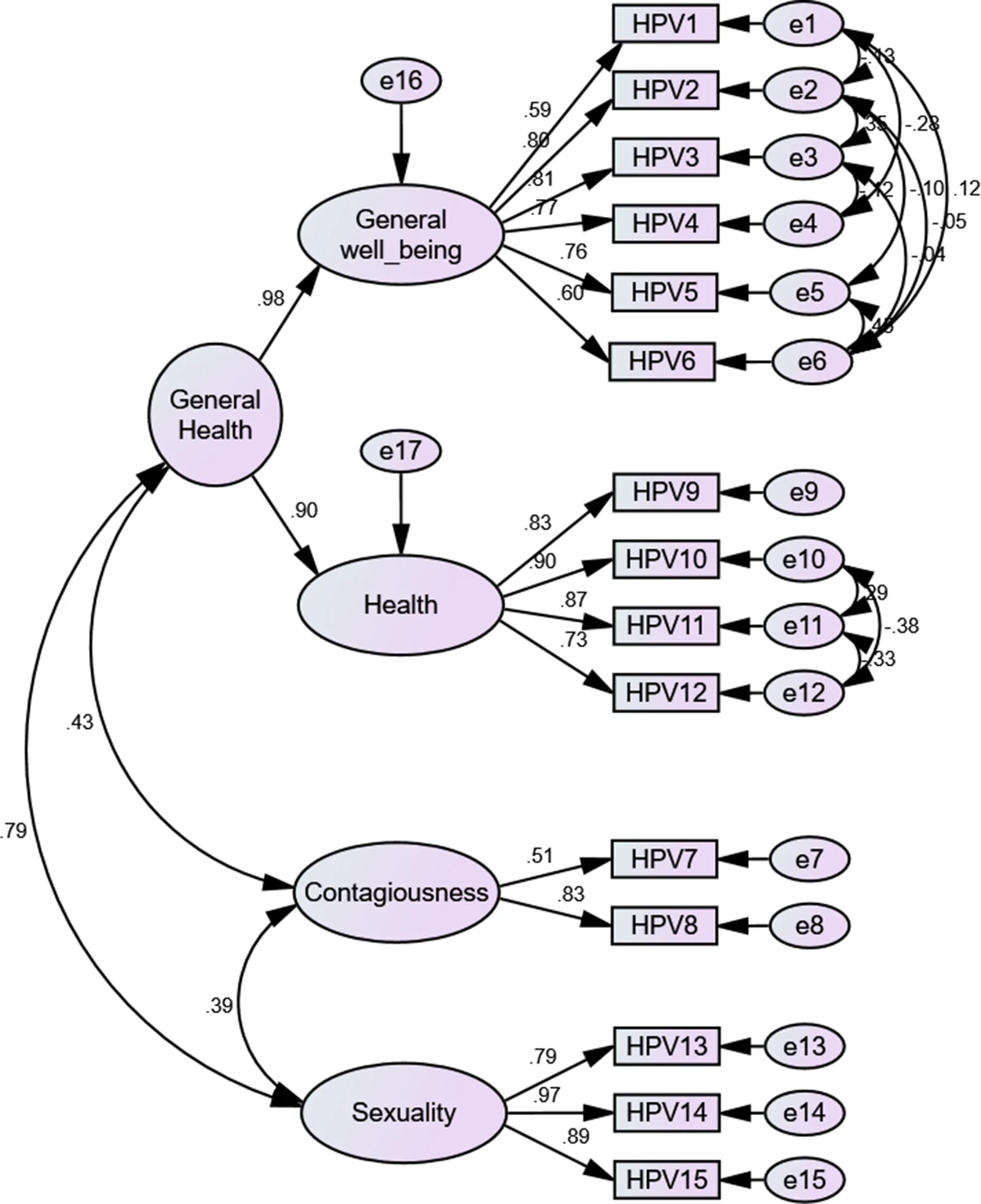
Confirmatory factor analysis of the modified second -order model of the Persian version of the HPV-QoL scale

Accordingly, a second-order confirmatory factor analysis was conducted (Fig. 3). The higher-order model demonstrated acceptable overall fit and improved conceptual parsimony (Table 3). Following model refinement, convergent and discriminant validity indices reached acceptable levels for all constructs except the contagiousness factor, which continued to demonstrate comparatively weaker convergent validity (Table 5).

**Table 5:** Convergent and discriminant validity indices of the Persian HPV-QoL

Model	Factor	CR	AVE	MSV	General well-being	Contagiousness	Health	Sexuality	
Revised first-order model	General well-being	0.868	0.526	0.774	1	-	-	-	
	Contagiousness	0.611	0.444	0.260	0.315	1	-	-	
	Health	0.888	0.667	0.774	0.783	0.364	1	-	
	Sexuality	0.916	0.786	0.608	0.716	0.269	0.654	1	
					**General health**	**Contagiousness**			**Sexuality**
Final second-order model	General health	0.938	0.885	0.624	1	-			-
	Contagiousness	0.631	0.476	0.185	0.353	1			-
	Sexuality	0.916	0.786	0.624	0.730	0.269			1

Abbreviations: CR = Composite Reliability; AVE = Average Variance Extracted; MSV = Maximum Shared Variance. Values below the diagonal represent interfactor correlations estimated from the confirmatory factor analysis

**Fig. 4:**
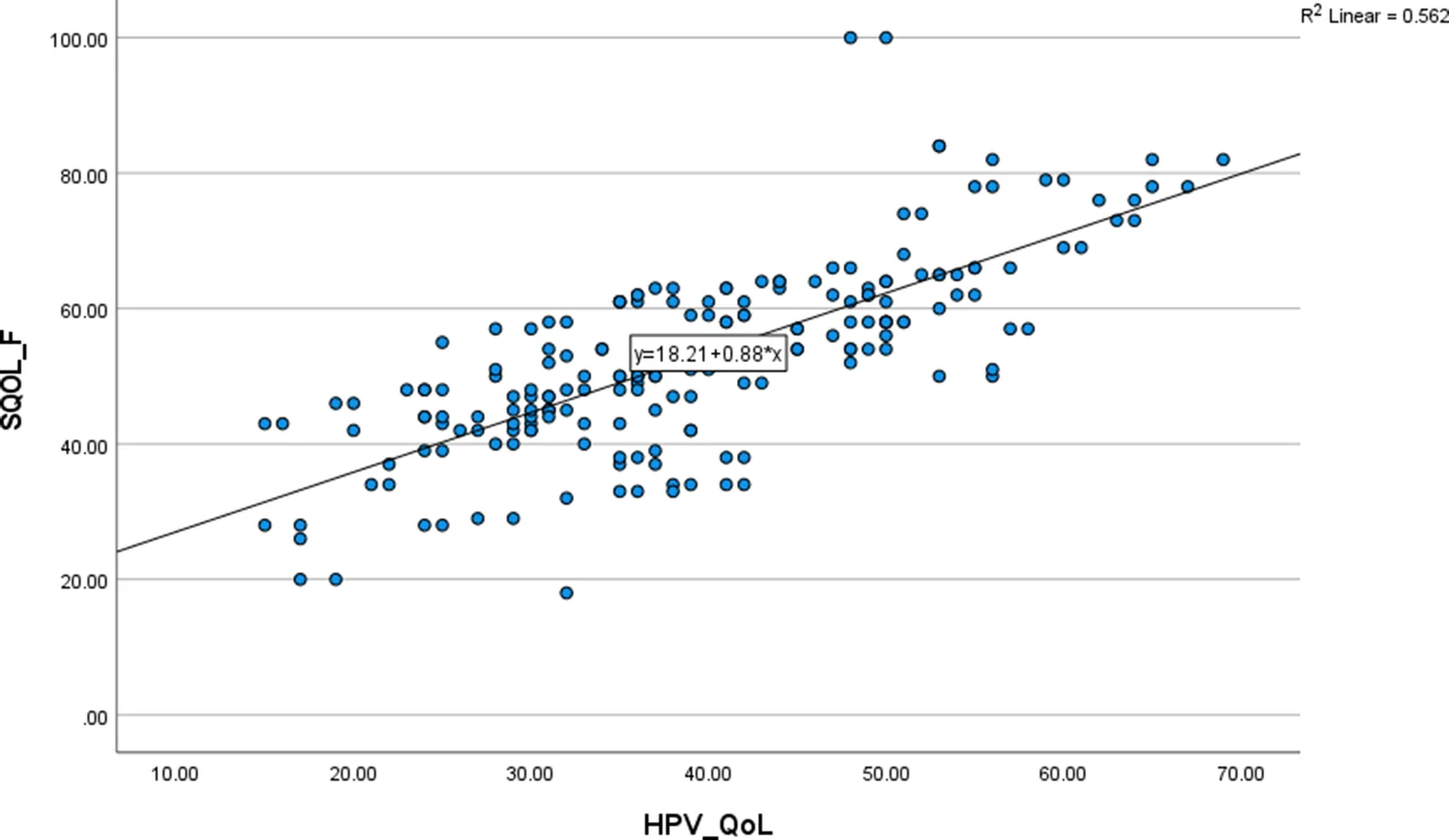
Correlation diagram between scores of Persian versions of HPV-QoL scale and SQOL-F

Criterion-related convergent validity was additionally assessed through correlation analysis between HPV-QoL scores and scores obtained from the Sexual Quality of Life-Female (SQOL-F) questionnaire. A strong positive correlation was observed between the two instruments (*r* = 0.751, *p* < 0.001), supporting satisfactory convergent validity of the Persian HPV-QoL (Fig. 4).

### Reliability, measurement error, and interpretability

The Persian HPV-QoL demonstrated satisfactory reliability and measurement stability. Internal consistency indices, including Cronbach’s alpha coefficients for the total scale and each subscale, are presented in Table 6 and were generally within acceptable ranges. Test–retest reliability analysis also demonstrated adequate temporal stability based on ICC values.

Absolute reliability was assessed using the SEM, which was 5.063. The SEM represents the amount of measurement error associated with a single observed score and provides an estimate of the precision of individual scores. Therefore, it should not be interpreted as the expected variation between repeated measurements. The reported SEM and MDC values were calculated based on the raw total score (15–75).

**Fig. 5:**
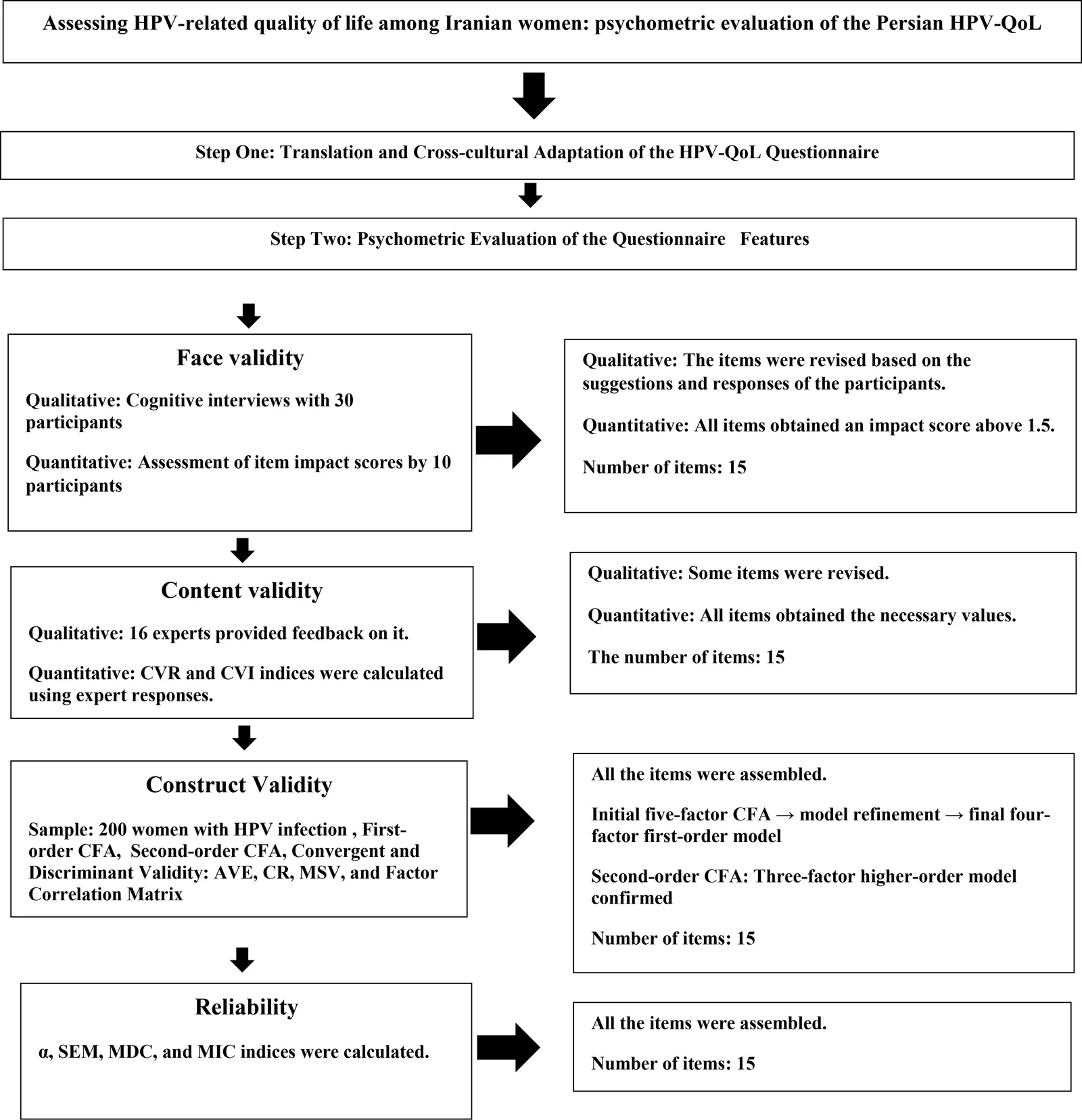
Workflow of the translation and psychometric evaluation of the Persian HPV-QoL

Measurement error and interpretability analyses further supported the measurement performance of the instrument. The MDC was 14.034, indicating that changes greater than this value can be interpreted as real changes beyond measurement error at the individual level with 95% confidence. The MIC was 1.249. In addition, floor and ceiling effects were minimal, with values of 0.01% and 0%, respectively, demonstrating an adequate score distribution and the absence of substantial response clustering at the extreme ends of the scale (Table 6). The overall workflow of the translation, cross-cultural adaptation, and psychometric evaluation of the Persian HPV-QoL is illustrated in Fig. 5.

**Table 6:** Reliability, measurement error, and interpretability indices of the Persian HPV-QoL

Factor	ICC	95% CI	Cronbach’s α	AIC	SEM	MDC	MIC
General well-being	0.815	0.211–0.945	0.870	0.525	2.383	6.605	0.842
Contagiousness	0.639	-0.101–0.880	0.595	0.426	0.810	2.245	0.712
Health	0.790	0.356–0.930	0.884	0.658	1.660	4.601	0.707
Sexuality	0.677	-0.007–0.893	0.909	0.772	1.843	5.109	0.681
Total HPV-QoL	0.833	0.423–0.947	0.929	0.462	5.063	14.034	1.249

Abbreviations: ICC = Intraclass Correlation Coefficient; AIC = Average Inter-item Correlation; SEM = Standard Error of Measurement; MDC = Minimal Detectable Change; MIC = Minimal Important Change

## Discussion

Human papillomavirus (HPV) infection is increasingly recognized as a condition associated not only with physical morbidity but also with substantial psychosocial and sexual burden that may adversely affect women’s health-related quality of life (HRQoL) [4, 7–16]. Women diagnosed with HPV frequently report anxiety, fear of cancer, shame, stigma, concerns regarding fertility and partner relationships, and distress associated with disclosure of the infection [8–14]. These psychosocial consequences may persist even in clinically asymptomatic women and may negatively influence healthcare-seeking behavior, adherence to follow-up care, and participation in screening programs [10, 11, 48–50]. Accordingly, culturally sensitive and psychometrically robust instruments are essential for accurately assessing the multidimensional impact of HPV infection on women’s lives.

The present study translated, culturally adapted, and psychometrically evaluated the Persian version of the HPV-QoL among Iranian women diagnosed with HPV infection. Overall, the findings demonstrated satisfactory validity, reliability, and interpretability of the Persian HPV-QoL. More importantly, the results provide insight into how HPV-related quality of life may be shaped by sociocultural context, particularly in settings where sexuality and sexually transmitted infections remain socially sensitive and highly stigmatized.

The findings of the content validity assessment demonstrated that all items achieved acceptable CVR and CVI values, indicating that the translated items were conceptually relevant, culturally appropriate, and sufficiently comprehensible for Iranian women [42, 51–54]. High agreement among experts further supports the adequacy of the adaptation process. These findings are broadly consistent with previous validation studies conducted in Spain and Brazil, suggesting that the core conceptual domains of HPV-related quality of life may remain relatively stable across different cultural settings [20, 21, 55]. Nevertheless, several linguistic and contextual modifications were required during the adaptation process to ensure semantic clarity and cultural acceptability. This issue is particularly important in the Iranian context, where perceptions of sexuality, sexually transmitted infections, and reproductive health are strongly influenced by sociocultural norms and stigma [8, 13, 14]. Accordingly, the adaptation process extended beyond literal translation and aimed to preserve conceptual equivalence while ensuring contextual relevance and cultural sensitivity. Construct validity analyses demonstrated that the Persian HPV-QoL retained an interpretable multidimensional structure.

CFA initially evaluated the original five-factor structure of the HPV-QoL. Following model refinement, the Persian version demonstrated a four-domain first-order structure encompassing General Well-being, Contagiousness, Health, and Sexuality. Although the Persian version retained the conceptual framework of the original instrument, the model refinement process indicated partial conceptual overlap between some domains, particularly General Well-being and Health. This refinement reflects the empirical structure observed in the present sample while preserving the conceptual content of the original instrument. These findings may indicate that, among Iranian women, aspects of emotional well-being and health-related perceptions are more closely interconnected than originally conceptualized. However, these observations should be interpreted cautiously, as they are based on model characteristics rather than direct assessment of sociocultural constructs.

One possible interpretation of this pattern is that it may reflect broader sociocultural characteristics of collectivist and socially conservative settings, where perceptions of sexually transmitted infections are often shaped by interpersonal relationships, family expectations, and moral norms [8, 13, 14]. Within such contexts, concerns about HPV transmission may extend beyond fear of infecting others to include worries about relationship stability, anticipated partner reactions, social rejection, and self-blame. Likewise, concerns related to sexuality may involve not only sexual functioning but also emotional intimacy, marital trust, body image, and perceived social identity [7, 9, 13].

Because these sociocultural factors were not directly assessed in the present study, these interpretations should be considered hypothesis-generating rather than definitive explanations. Nevertheless, they underscore the importance of considering cultural context when interpreting HPV-related quality of life and support the need for future studies that directly examine these sociocultural influences.

Confirmatory factor analysis further supported the structural validity of the Persian HPV-QoL, with the revised model demonstrating acceptable overall fit across multiple goodness-of-fit indices [34–36, 56–58]. In addition, convergent and criterion validity analyses demonstrated theoretically coherent associations between HPV-QoL domains and measures of sexual quality of life and psychological well-being. These findings are consistent with previous evidence indicating that HPV infection may substantially affect emotional functioning, sexual well-being, interpersonal relationships, and self-perception [9, 16, 22, 23, 48]. The strong correlation observed between the HPV-QoL and SQOL-F further supports the construct validity of the Persian version and reinforces the multidimensional nature of HPV-related quality of life.

The reliability analyses also demonstrated satisfactory psychometric performance. The Persian HPV-QoL showed strong internal consistency and acceptable temporal stability for the total scale. Although the contagiousness domain demonstrated comparatively weaker convergent validity and lower internal consistency than the other domains, its psychometric performance remained within acceptable ranges for exploratory and descriptive research [40]. Importantly, this comparatively weaker performance may itself reflect the contextual complexity of HPV-related transmission concerns. Perceptions regarding contagion are likely influenced not only by medical knowledge but also by cultural beliefs, stigma, relationship dynamics, fear of disclosure, and perceived partner responses. Consequently, concerns related to transmissibility may be more heterogeneous and context-dependent than other dimensions of HPV-related quality of life. In addition to representing a psychometric limitation, this finding may also reflect transmission-related distress shaped by sociocultural and interpersonal experiences that vary across individuals and cultural settings.

An important contribution of the present study lies in the comparative positioning of the HPV-QoL relative to other HPV-specific quality-of-life instruments available in Persian. Existing instruments such as the HPV Impact Profile (HIP) and EQUALI-HPV have demonstrated acceptable psychometric properties; however, they are comparatively longer and may impose greater respondent burden [12, 59]. In contrast, the HPV-QoL offers a relatively brief and clinically feasible assessment while still capturing multiple dimensions of HPV-related quality of life, including emotional well-being, sexuality, and concerns regarding transmission and health. This brevity may improve applicability in busy clinical settings and large-scale epidemiological studies, particularly in contexts where lengthy questionnaires may reduce response quality or participant engagement. Furthermore, the availability of international normative data for the HPV-QoL enhances opportunities for cross-cultural comparison and international collaborative research (21). The present findings also have important clinical implications. Women diagnosed with HPV frequently experience psychosocial distress that may remain underrecognized during routine clinical care [4, 8, 13, 16]. In many healthcare settings, management of HPV primarily focuses on diagnostic surveillance and physical disease outcomes, whereas emotional distress, stigma, sexual concerns, and relationship-related difficulties receive comparatively limited attention [13, 48–50, 60]. The Persian HPV-QoL may therefore serve as a clinically useful tool for identifying women experiencing substantial HPV-related psychosocial burden and for evaluating counseling, psychosexual, and educational interventions. More broadly, integrating culturally sensitive HRQoL assessment into HPV-related care may facilitate more patient-centered approaches to reproductive and sexual healthcare.

Several limitations should nevertheless be acknowledged. First, the use of self-report measures may have introduced recall bias and social desirability bias, particularly in sexuality-related domains given the sociocultural sensitivity of these issues. Second, participants were recruited from clinical centers within one geographical region of Iran, which may limit the generalizability of the findings to other populations and sociocultural settings. Third, although the overall psychometric properties of the Persian HPV-QoL were satisfactory, the Contagiousness domain did not fully meet the recommended criterion for convergent validity, and this should be considered when interpreting the findings. Fourth, detailed clinical characteristics, including HPV genotype, cytology or lesion status, time since diagnosis, treatment history, sexual activity, and other relevant clinical variables, were not collected and therefore could not be examined for their potential influence on HPV-related quality of life. Finally, although the present study provides preliminary evidence supporting the validity and reliability of the instrument, further studies with independent samples are needed to confirm the structural stability of the questionnaire, evaluate cross-cultural measurement equivalence, and establish responsiveness and clinically meaningful interpretation of score changes over time. Furthermore, because the MIC values were lower than the corresponding MDC values, small but clinically meaningful changes may not always be distinguishable from measurement error at the individual level. Consequently, additional longitudinal studies are warranted to further evaluate the responsiveness of the Persian HPV-QoL and to establish clinically meaningful thresholds for interpreting changes in scores over time.

## Conclusion

In conclusion, the Persian version of the HPV-QoL provides preliminary evidence of satisfactory validity and reliability for assessing HPV-related quality of life among Iranian women. The findings support the applicability of the instrument within the Iranian cultural context; however, further studies are needed to establish independent structural confirmation, cross-cultural measurement equivalence, responsiveness, and clinically meaningful interpretation of score changes over time. In addition, the observed factor structure may partly reflect sociocultural influences, including stigma, interpersonal relationships, and culturally mediated perceptions of sexuality and contagion. Because these factors were not directly measured in the present study, these interpretations should be considered hypothesis-generating rather than definitive conclusions. Overall, the findings underscore the importance of considering cultural context when assessing HPV-related quality of life and provide a foundation for future research on culturally sensitive psychosocial assessment and interventions in women with HPV infection.

## Data Availability

No datasets were generated or analysed during the current study.

## Abbreviations

HPV: Human Papillomavirus
QoL: Quality of Life
HRQoL: Health-Related Quality of Life
STIs: Sexually Transmitted Infections
HIP: HPV Impact Profile
CECA-10: Cuestionario Específico en Condilomas Acuminados
CSFQ: Changes in Sexual Functioning Questionnaire
CVR: Content Validity Ratio
CVI: Content Validity Index
CFA: Confirmatory Factor Analysis
SQOL-F: Sexual Quality of Life–Female
KMO: Kaiser–Meyer–Olkin
RMSEA: Root Mean Square Error of Approximation
GFI: Goodness-of-Fit Index
IFI: Incremental Fit Index
PCFI: Parsimony Comparative Fit Index
AGFI: Adjusted Goodness-of-Fit Index
AVE: Average Variance Extracted
CR: Composite Reliability
MSV: Maximum Shared Variance
SEM: Standard Error of Measurement
MDC: Minimal Detectable Change
MIC: Minimal Important Change
AIC: Average Inter-item Correlation
ICC: Intraclass Correlation Coefficient
